# Polyion Hydrogels of Polymeric and Nanofibrous Carboxymethyl Cellulose and Chitosan: Mechanical Characteristics and Potential Use in Environmental Remediation

**DOI:** 10.3390/gels10090604

**Published:** 2024-09-23

**Authors:** Taisei Kawate, Yehao Wang, Kayee Chan, Nobuyuki Shibata, Yuya Doi, Yuichi Masubuchi, Anatoly Zinchenko

**Affiliations:** 1 Graduate School of Environmental Studies, Nagoya University, Nagoya 464-8601, Japan; 2 Nagoya Municipal Industrial Research Institute, Nagoya 456-0058, Japan; shibata.nobuyuki@nmiri.city.nagoya.jp; 3 Graduate School of Engineering, Nagoya University, Nagoya 464-8601, Japan; ydoi@yz.yamagata-u.ac.jp (Y.D.); mas@mp.pse.nagoya-u.ac.jp (Y.M.); 4 Graduate School of Organic Materials Science, Yamagata University, Yonezawa 992-8510, Japan

**Keywords:** nanofibers, interpolyelectrolyte complex, hydrogel, rheology, metal ion adsorption

## Abstract

Recently, cellulose and other biomass nanofibers (NFs) have been increasingly utilized in the design of sustainable materials for environmental, biomedical, and other applications. However, the past literature lacks a comparison of the macromolecular and nanofibrous states of biopolymers in various materials, and the advantages and limitations of using nanofibers (NF) instead of conventional polymers are poorly understood. To address this question, hydrogels based on interpolyelectrolyte complexes (IPECs) between carboxymethyl cellulose nanofibers (CMCNFs) and chitosan (CS) were prepared by ele+ctrostatic cross-linking and compared with the hydrogels of carboxymethyl cellulose (CMC) and CS biopolymers. The presence of the rigid CMCNF altered the mechanism of the IPEC assembly and drastically affected the structure of IPEC hydrogels. The swelling ratios of CMCNF-CS hydrogels of ca. 40% were notably lower than the ca. 100–300% swelling of CMC-CS hydrogels. The rheological measurements revealed a higher storage modulus ($G^{\prime }$) of the CMCNF-CS hydrogel, reaching 13.3 kPa compared to only 3.5 kPa measured for the CMC-CS hydrogel. Further comparison of the adsorption characteristics of the CMCNF-CS and CMC-CS hydrogels toward Cu^2+^, Cd^2+^, and Hg^2+^ ions showed the slightly higher adsorption capacity of CMC-CS for Cu^2+^ but similar adsorption capacities for Cd^2+^ and Hg^2+^. The adsorption kinetics obeyed the pseudo-second-order adsorption model in both cases. Overall, while the replacement of CMC with CMCNF in hydrogel does not significantly affect the performance of such systems as adsorbents, CMCNF imparts IPEC hydrogel with higher stiffness and a frequency-independent loss ($G^{\prime \prime }$) modulus and suppresses the hydrogel swelling, so can be beneficial in practical applications that require stable performance under various dynamic conditions.

## 1. Introduction

The design of polymer materials based on simple electrostatic interactions between polycations and polyanions, both biological and synthetic, has received considerable attention due to its simplicity and robustness in allowing the facile control of the structure of such systems to construct functional materials [1,2]. The mixing of oppositely charged polymers such as carboxymethyl cellulose (CMC) and chitosan (CS) results in the formation of polyion or interpolyelectrolyte complexes (IPECs) [3,4,5]. The controlled assembly of IPECs was used to prepare IPEC nanoparticles [6], two-dimensional multilayer films [7], three-dimensional networks of hydrogels [8,9], and other materials. IPEC hydrogel materials have been successfully employed in the drug delivery [10], wound healing [11], and wastewater treatment [12,13] fields.

Biopolymer-based materials are widely applied in the environmental field for the removal of various pollutants such as heavy metals [14]. Heavy metals such as Cu, Cd, and Hg are major pollutants in freshwater reserves because of their toxic, non-biodegradable, and persistent nature. Cellulose and chitosan derivatives show notable affinity for heavy metal ions; therefore, they are utilized to fabricate a vast number of adsorbents for water treatment. In particular, IPEC hydrogels of CMC and CS are utilized for the removal of heavy metal ions and other pollutants such as industrial dyes and pharmaceuticals [13,15,16,17]. Furthermore, IPEC networks are used to impart an adsorption function to other composite materials. For instance, the incorporation of IPEC into a soil matrix was shown to suppress the leaching of heavy metals from contaminated soil [18].

Biomass-derived nanofibers (NFs) emerged as a new sustainable material and gained significant interest in material design [19]. Cellulose nanofiber (CNF), for instance, possesses unique mechanical characteristics such as a tensile strength (σ) of up to 8 GPa, crystal modulus of about 140 GPa, and density of 1.6 g/cm^3^ [20], which make it5–7 times stronger than steel despite having only 1/5 of steel’s density. The strength, flexibility, and specific nano-structuration of CNF and its derivatives are widely utilized in optoelectronics, antibacterial coatings, mechanically reinforced polymer composites, tissue scaffolds, drug delivery, biosensors, energy storage, catalysis, etc. [21]. Production technologies of NFs from other biomass materials such as chitosan and chitin and their applications have also been actively developed [22,23]. Recently, CNF has been increasingly utilized in materials for environmental remediation, including water purification of heavy metals [24,25]. CMF-containing hydrogel materials have also been reported [26,27,28].

Biomass NF, having the aforementioned unique properties, represents an attractive alternative to conventional polymeric materials, and the substitution of polymeric components in IPEC networks with nanofibrous counterparts may significantly affect the properties of such systems. However, there has been little discussion in the literature on a comparison between polymeric and nanofibrous materials of similar composition. Herein, we utilized carboxymethylcellulose nanofibers (CMCNFs) and chitosan (CS) to prepare IPEC-type hydrogel to compare its formation conditions, rheological behavior, and metal ion adsorption characteristics with those of an analogous hydrogel of macromolecular CMC and CS.

## 2. Results and Discussion

### 2.1. Formation Mechanism and Structure of CMCNF-CS and CMC-CS Hydrogels

**Figure 1:**
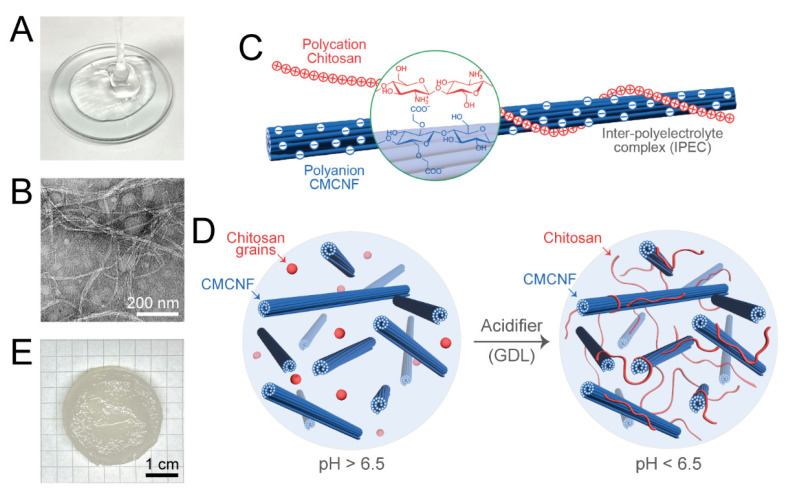
(**A**) Photographic image of 2% (*w*/*w*) aqueous dispersion of CMCNF. (**B**) TEM image of CMCNF stained with UranyLess staining agent. (**C**) Schematic illustration of the formation of IPEC from cationic CS and anionic CMCNF by electrostatic interactions. (**D**) Schematic illustration of the mechanism of the CMCNF-CS hydrogel formation by a gradual decrease in solution pH using a GDL acidifier. (**E**) Photographic image of a typical CMCNF-CS hydrogel after swelling in pure water.

CMC nanofiber (CMCNF) dispersions prepared by water-jet defibrillation are highly viscous solutions even at concentrations of a few percent (Figure 1A). CMCNF dispersions contain CMC nanofibers of ca. 10 nm in diameter and several micrometers in length (Figure 1B). Due to the hydrophilic character of CMCNF with anionic carboxy groups, CMCNF shows excellent dispersion properties with no aggregation in aqueous media according to TEM images (Figure 1B). CMCNF and CS can form ionic complexes through electrostatic interactions between the carboxy groups of CMCNF and the amino groups of CS (Figure 1C); however, direct mixing of a CMCNF dispersion and CS solution results in the formation of poorly controlled coacervates. Therefore, CMCNF-CS hydrogels were prepared by the method developed by Morikawa et al. [9], illustrated in Figure 1D, based on the pH dependence of CS solubility. Hydrogels of CMC and CS were prepared in the same manner. CS has a pK_a_ of 6.5 and does not notably dissolve in either CMCNF or CMC solutions of neutral pH. The addition of glucono-*δ*-lactone (GDL) to either mixture results in the lowering of the pH and the consequent dissolution of CS due to the protonation of CS amines. Consequently, the amino groups of the dissolved chitosan and the carboxyl groups of CMCNF or CMC interact to form an electrostatic network of physically cross-linked hydrogel, which appeared as a monolith, translucent material (Figure 1E). It was noted that while gelation in the CMC-CS system occurred at the initial c(CMC) above 1% (*w*/*w*), the dispersion of CMCNF with a concentration of >2% was required to prepare a stable hydrogel.

**Figure 2:**
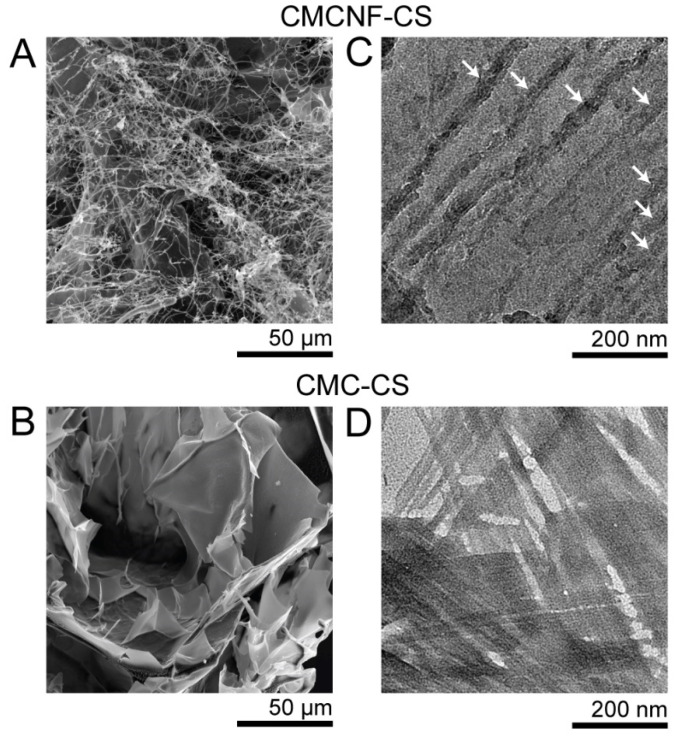
(**A**,**B**) Typical SEM images of CMCNF-CS (**A**) and CMC-CS (**B**) hydrogel fragments after freeze-drying. (**C**,**D**) Typical TEM images of CMCNF-CS (**C**) and CMC-CS (**D**) hydrogel fragments after freeze-drying. Arrows in (**C**) point to the fibrils of CMCNF-CS complexes.

The FTIR spectra of the freeze-dried CMC-CS and CMCNF-CS hydrogels (Figure S1) were similar except for the band around 1750 cm^−1^, assigned to the C=O stretching of the -COOH group, which was lower for the CMCNF-CS hydrogel. This difference can be attributed to the lower carboxylation degree in the CMCNF than in the CMC. The SEM image of freeze-dried CMCNF-CS hydrogel (Figure 2A) shows that the hydrogel is mainly composed of microfibrils of a 100–200 nm diameter but also contains a minor part of film-like structures. In contrast, the IPEC hydrogels of CMC and CS are predominately composed of very large films (Figure 2B) that appear thin at the nanoscale (Figure 2D), which is in agreement with previous studies [9,18].

Compared to the flexible chains of CMC macromolecules, CMCNF has a rigid rod morphology. Furthermore, the diffusion rates of the macromolecules of CMC are drastically faster compared to those of nanofibers [29]. As a result, IPEC formation between CMC and chitosan is considered to involve the fast delivery of gradually dissolving CS to CMC, which self-assemble planarly into films to form a large number of ionic bonds. In contrast, the assembly of a film structure from CMCNF and CS has several constraints. The appearance of an interface in CMCNF drives the adsorption of CS on the CMCNF surface. The slow and anisotropic diffusion of nanofibers prevents the aligning of multiple fibers to form a lamellar structure. Therefore, the morphology of CMCNF-CS hydrogel appears as a randomly oriented anionic NF or their bundles cross-linked with cationic macromolecules of CS. In agreement with the proposed mechanism, the TEM images of freeze-dried CMCNF-CS hydrogel (Figure 2C) show individual fibrils with a thickness of 20–30 nm, indicating the formation of IPEC fibers containing a few CMCNFs with a ca. 10 nm diameter.

### 2.2. Comparison of Swelling and Polymer Elution Behavior of CMCNF-CS and CMC-CS Hydrogels

A series of CMCNF-CS and CMC-CS hydrogels were prepared by dispersing 30 mg, 90 mg, and 120 mg of CS in 3 mL of 2% CMCNF or CMC solution to address the effect of the polyanion to polycation ratio. Hereafter, the difference in the amount of added CS is shown in the abbreviation of the corresponding hydrogel by adding the weight of the chitosan at the end, for example, CMCNF-CS30 for the hydrogel prepared using 30 mg of CS. The addition of CS at an amount smaller than 30 mg or larger than 120 mg resulted in either no gelation, or the gelated system was unstable and dispersed upon transferring to water, obviously due to the domination of the repulsive interaction between polyions presented in excess.

**Figure 3:**
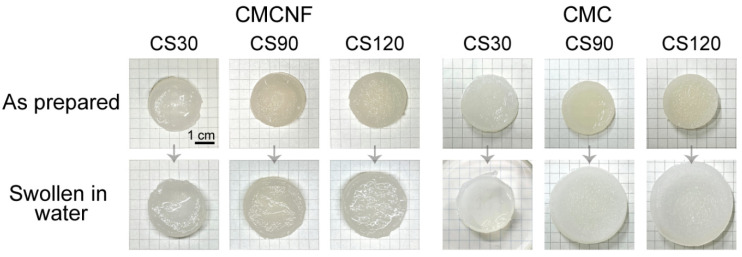
Photographic images of CMCNF-CS and CMC-CS hydrogels as prepared (**top**) and after equilibration in the distilled water (**bottom**).

The washing and equilibration of the hydrogels after preparation in a large amount of pure water resulted in the swelling of the hydrogels as well as a release of a part of the unreacted polymers. Photographic images of the as-prepared hydrogels and hydrogels after washing and equilibration in the distilled water are shown in Figure 3. Both types of hydrogels exhibited higher swelling degrees at higher ratios of added CS, which was due to the increasing role of repulsion interactions in the hydrogels containing an excess of cationic CS (Figure 3, bottom). However, the swelling rates of the CMC-CS hydrogels of up to 272% were notably higher than those of the CMCNF-CS hydrogels, which reached only 43% (Table 1). This difference suggests that in contrast to the hydrogel composed of flexible macromolecules of CMC and CS, the presence of rigid CMCNF in the hydrogel structure imposes conformational constraints on the polymer network that suppress hydrogel swelling.

Table 1 shows the elution rates of the polymers from the CMCNF-CS and CMC-CS gels based on gravimetric analysis. The polymer elution rates tended to increase for both types of hydrogels due to the increasing contribution of non-bound CS added in excess. Interestingly, despite the low available negative charge on CMCNF compared to that of macromolecular CMC taken at the same concentration, the elution rates were lower in the case of the CMCNF-CS hydrogels. When the amount of CS was about one-half of the CMCNF weight (CMCNF-CS30), no release of polymers was measured. Neither a change in the swelling ratio nor the elution of polymers from any type of prepared hydrogels was measured after two equilibrations in the deionized water, indicating the structural stability of the polymer network in the hydrogels.

### 2.3. Rheological Properties of CMCNF-CS and CMC-CS Hydrogels

**Figure 4:**
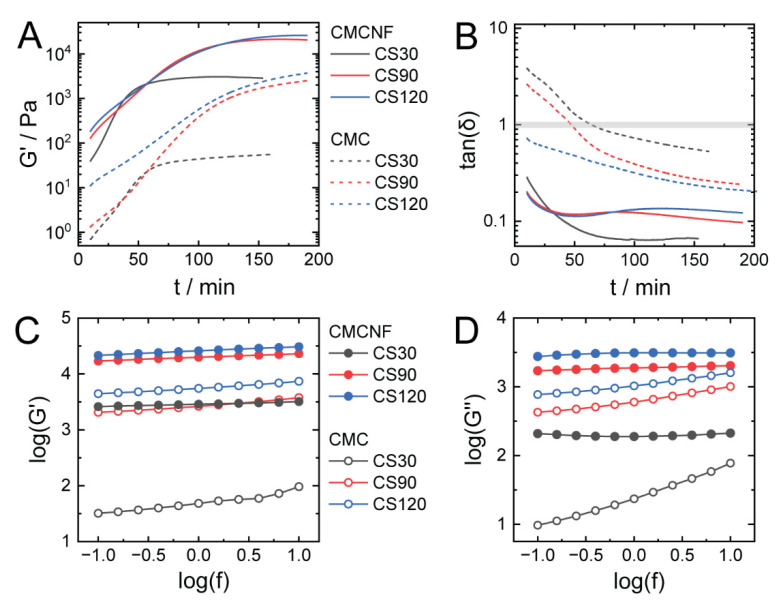
(**A**) Time dependences of storage modulus ($G^{\prime }$) during gelation of CMC-CS and CMCNF-CS hydrogels containing different amounts of CS. (**B**) Time dependence of tan(δ) of CMC-CS and CMCNF-CS hydrogels. (**C**,**D**) Frequency dependences of storage modulus $G^{\prime }$ (**C**) and loss modulus $G^{\prime \prime }$ (**D**) of CMC-CS and CMCNF-CS hydrogels.

Rheological measurements were performed to quantitatively compare the mechanical characteristics of CMCNF-CS and CMC-CS hydrogels prepared using different amounts of CS. To monitor the gelation process, elastic modulus ($G^{\prime }$) and viscous modulus ($G^{\prime \prime }$) measurements were conducted after the initial mixing of CS and GDL powders in CMC solution or CMCNF dispersion for 10 min. The kinetic profiles of CMCNF-CS and CMC-CS gelation (Figure 4A) showed an increase in the storage modulus ($G^{\prime }$) over time due to the gelation associated with the increase in hydrogel stiffness. The gelation of both types of hydrogels in the presence of 30 mg of CS was characterized by a gradual increase in $G^{\prime }$, followed by a plateau. The gelation of CMCNF-CS was faster and accomplished in 30 min, in comparison to the 50 min required for the gelation of the CMC-CS hydrogel. At larger amounts of added CS, the gelation times were considerably longer in both cases but similar for samples with 90 mg and 120 mg of CS. Longer gelation times can be attributed to lower rates of CS dissolution and diffusion in systems with higher total concentrations of polymers. Again, the gelation of CMCNF in the presence of CS was faster than that of polymeric CMC and reached equilibrium after 2 h, while a moderate increase in $G^{\prime }$ in the CMC-CS system was still observed even after 150 min. This difference may indicate slow structural rearrangements in the inner structure of IPEC hydrogel films.

At the end of the gelation process, the storage modulus of the CMCNF-CS hydrogels was 5–30 times higher than that of the CMC-CS hydrogels, yet this increase could have partly been a result of the higher initial storage moduli of the CMCNF dispersion compared with that of the CMC solutions having a ca. 1 order of magnitude difference in $G^{\prime }$. The effect of the CS amount on the final $G^{\prime }$ values was the same in both systems. A significant increase in storage modulus was observed for the CS90 samples compared to CS30, while there was almost no difference between CS90 and CS120 specimens. The time dependences of $G^{\prime }$ for all studied hydrogels were well-fitted ($R^{2}>0.99$) with a modified Hill’s Equation (1), as described in detail elsewhere [30], and the highest steady-state storage modulus was estimated as 13.3 kPa for the CMCNF-CS120 hydrogel and as 3.5 kPa for the CMC-CS120 hydrogel.

(1) $$G^{\prime }(t)={G^{\prime }}_{\infty }\frac{t^{n}}{t^{n}+k^{n}}$$

The time-dependent changes of the loss tangent ($tan(\delta )=G^{\prime }/G^{\prime \prime }$) in each system are shown in Figure 4B. At the end of gelation, all types of hydrogels showed tan(δ)<1, indicating the solid-like behavior of the hydrogels. While in the CMC-CS system, a clear sol-gel transition from the vicious (tan(δ)>1) to the elastic (tan(δ)<1) state was observed during gelation, the loss tangent of CMCNF-CS systems was below 1 at the beginning of measurements, indicating elastic behavior. This can be explained by the formation of a gel-like structure as a result of the percolation of CMCNF fibers and weak inter-fiber interactions such as hydrogen bonding. Nevertheless, similar to the CMC-CS system, the interaction of CMCNF with the dissolved CS and the formation of IPEC complexes resulted in a further decrease in tan(δ). Interestingly, the effect of the added CS amount on the tan(δ) of CMC-CS and CMCNF-CS was reversed: larger amounts of added chitosan resulted in more elastic CMC-CS gels, while the elasticity of CMCNF-CS was highest when smaller amounts of chitosan were added. The fact that larger amounts of chitosan result in more elastic CMC-CS gels while smaller amounts lead to higher elasticity in CMCNF-CS gels may reflect the internal structure of the gel affecting the balance between $G^{\prime }$ and $G^{\prime \prime }$.

Frequency sweep tests of both types of hydrogels, except CMC-CS30, at frequencies in the range of between 0.1 Hz and 10 Hz showed a weak increase in $log(G^{\prime })$ with the increase in frequency (Figure 4C), indicating that the examined systems exhibited typical responses of gels. The CMC-CS30 hydrogel showed a slightly stronger frequency-dependent increase in $log(G^{\prime })$. In contrast, the frequency dependences of the loss modulus, $G^{\prime \prime }$, were clearly distinct between the series of CMC-CS and CMCNF-CS hydrogels (Figure 4D). The hydrogel containing nanofibers exhibited essentially frequency-independent behavior in the whole range of frequencies. The CMC-CS hydrogels showed a moderate (CMC-CS90 and CMC-CS120) or even strong (CMC-CS30) increase in $log(G^{\prime \prime })$, indicating the domination of the viscous component of the material over the elastic component, meaning that CMC-CS hydrogel deforms more irreversibly and dissipates more energy as the deformation speed increases.

### 2.4. Adsorption of Heavy Metal Ions on CMCNF-CS and CMC-CS Hydrogels

Various materials prepared from biopolymers, including hydrogels, have been intensively studied for separation and adsorption applications [13,15,16,17]. Among the targeted pollutants, heavy metal ions having high toxicity have been some of the most studied because cellulose, chitosan, and other widely used biopolymers have a high density of functional groups that can serve as binding sites for metal ions.

**Figure 5:**
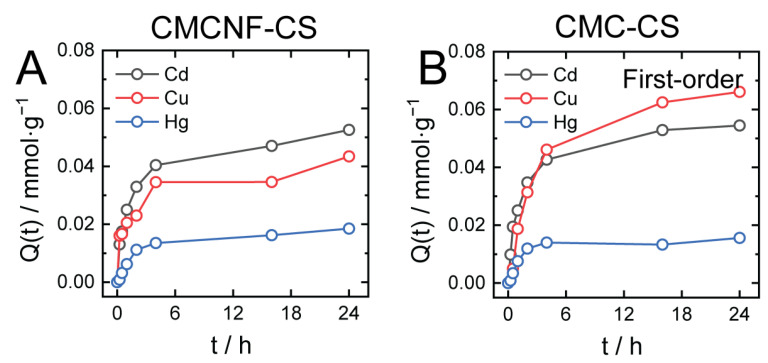
Time-dependent adsorption of Cu^2+^, Cd^2+^, and Hg^2+^ ions by CMCNF-CS90 (**A**) and CMCNF-CS90 (**B**) hydrogels expressed in mmol/g units.

To compare the potential of CMC-CS90 and CMCNF-CS90 hydrogels for the removal of Cu^2+^, Cd^2+^, and Hg^2+^ cations, preliminary batch adsorption tests were conducted. IPEC hydrogels were placed into aqueous solutions of Cu^2+^, Cd^2+^, and Hg^2+^ of 10 ppm concentrations, and changes in metal ion concentration were measured by the ICP-AES method. Adsorption experiments were conducted at pH 7–7.5 because previous studies have shown that the most efficient adsorption occurs at a solution pH of around 7 [15]. The adsorption kinetics data for both hydrogels are shown in Figure 5. Both hydrogels showed fast kinetics for all types of studied metal ions within 4 h, achieving 70–80% of the maximal removal rates, while further adsorption was slower, which could be attributed not only to the slow adsorption kinetics but also to changes in the hydrogel structure due to metal ion adsorption (Figure 5). In agreement with earlier studies on the related systems [15], the adsorption of metal ions requires considerable time, in the order of 10 h, which is attributed to the slow diffusion of metal ions inside the hydrogel as well to the divalent-ion-induced rearrangements in the hydrogel structure affecting hydrogel adsorption capacity. The adsorption process was somewhat faster for the CMCNF-CS hydrogel than for CMC-CS, which might be related to the structural arrangements in CMC-CS induced by the divalent cations. The adsorption of Hg^2+^ metal ions reached equilibrium faster than of the other metal ions, within about 5 h, which might have been due to the difference in the binding sites of the studied metal ions.

Figure 5 shows that, on mmol/g bases, both gels adsorbed the least amount of Hg at similar rates in both cases. CMCNF-CS showed a higher adsorption capacity toward Cd compared to Cu, while the CMC-CS hydrogel showed a higher adsorption capacity toward Cu. Providing that the amino group of CS is the primary binding site for Cu ions in the hydrogels, according to previous studies [31], the higher adsorption capacity of the CMC-CS hydrogel suggests a higher availability of NH_2_ groups for binding with metal ions. On the mg/g scale, the adsorption capacities of both hydrogels were in the range of 4–6 mg/g, which is generally low compared to, for instance, CMC-CS hydrogel cross-linked by irradiation [17]. The lower adsorption capacities of IPEC hydrogels are explained by the consumption of a part of the CMC, CMCNF, and CS functional groups (-COOH and -NH_2_) for electrostatic cross-linking, which reduces the number of possible binding sites for metal ions.

To gain further insight into the adsorption characteristics of CMCNF-CS and CMC-CS hydrogels, the kinetic data were fit with pseudo-first- and pseudo-second-order adsorption kinetics models (Figure S2). The fitting parameters based on the results of adsorption experiments (Table 2) indicate that in most cases the adsorption was in better agreement with the second-order adsorption kinetics model. The better fitting of the data by the pseudo-second-order adsorption kinetics model demonstrates that the adsorption of metal ions to all types of hydrogels is chemical. In the case of metal ion removal by the CMCNF-CS hydrogel, the rate constants $k_{2}$ obtained from the pseudo-second-order model in the order of Cd > Cu > Hg indicated a faster uptake by CMCNF-CS hydrogel in the same order, which may have resulted from the different affinities between metal ions and hydrogel. Overall, the adsorption kinetics and adsorption capacities of CMCNF-CS and CMC-CS hydrogels for a certain metal ion were similar, indicating that the substitution of CMC by CMCNF in the hydrogel does not significantly change the hydrogel adsorption characteristics.

## 3. Conclusions

We have successfully prepared an IPEC hydrogel of CMCNF and CS based on their interpolyelectrolyte complex formed by the gradual ionization of CS and electrostatic interaction with CMCNF. The CMCNF-CS hydrogel materials derived from biomass polymers represent sustainable and renewable composite materials that are environmentally benign and “green” by design. In comparison to a hydrogel prepared from a pair of polymeric CMC and CS, CMCNF-CS hydrogel shows suppressed swelling and more solid-like behavior. Furthermore, CMCNF-CS hydrogels demonstrate a frequency-independent loss ($G^{\prime \prime }$) modulus. The suppressed swelling, higher stiffness, and consistent mechanical properties of NF-containing hydrogels across a range of deformation rates offer reliable damping, stability, and structural integrity properties, making NF-containing hydrogels attractive for use in medical devices, tissue engineering, and controlled drug delivery. On the other hand, the metal ion adsorption characteristics of both hydrogels are similar, suggesting that IPEC hydrogels containing NF can be applied equally well for adsorption/desorption applications while providing additional benefits associated with their enhanced mechanical characteristics.

## 4. Materials and Methods

### 4.1. Materials

For these experiments, 2 wt% dispersions of carboxymethyl cellulose nanofibers (CMCNFs) were purchased from Sugino Machine Ltd. (Toyama, Japan). Carboxymethyl cellulose sodium salt (250,000 g/mol), and low-molecular-weight chitosan (50,000–190,000 g/mol, 75–85% deacetylation degree) were purchased from Sigma-Aldrich (St. Louis, MO, USA). D-(+)-glucono-*δ*-lactone (GDL) was purchased from Tokyo Chemical Industry Co. (Tokyo, Japan). Copper standard solution (1000 ppm), cadmium standard solution (100 ppm), and mercury standard solution (100 ppm) were purchased from Wako Fujifilm (Osaka, Japan). Ultrapure water purified by a Purelab Chorus 1 Life Science (ELGA LabWater, Buckinghamshire, UK) apparatus was used in all experiments.

### 4.2. Methods

#### 4.2.1. Rheological Measurements

Gelation of the hydrogels was studied by placing sample mixtures in a 1 mm gap between two parallel plates 50 mm in diameter, and measurements were performed on an MCR 301 rheometer (Anton Paar, Austria) in oscillatory sweep mode at a frequency of f = 1 Hz and strain amplitude of γ = 1.0% at 25 °C. The loss tangent (tan(δ)) was calculated from G′ and G″ using the equation below:

(2) $$tan(\delta )=G^{\prime \prime }/G^{\prime }$$

where $G^{\prime }$ and $G^{\prime \prime }$ are the elastic and viscous modulus, respectively.

Frequency dependencies of the shear moduli of the hydrogels placed in a 1 mm gap between two parallel plates of 50 mm in diameter were measured in a frequency range of 0.1 to 10 Hz at strain amplitude γ = 1.0%.

#### 4.2.2. Scanning Electron Microscopy (SEM)

SEM observations of freeze-dried (Eyela FDU-1200, Tokyo Rikakikai Co., Ltd, Tokyo, Japan) hydrogels after carbon coating were performed at room temperature on a JSM-6610 microscope (JEOL Ltd., Tokyo, Japan) at an acceleration voltage of 15 kV.

#### 4.2.3. Transmission Electron Microscopy (TEM)

Freeze-dried CMCNF-CS hydrogels were cut into small pieces by scalpel, mounted on a lacey carbon-coated copper grid (Alliance Biosystems, Nagoya, Japan), and directly observed on a JEM-2100 Plus microscope (JEOL Ltd., Tokyo, Japan) at 200 kV acceleration voltage at room temperature.

#### 4.2.4. Inductively Coupled Plasma Atomic Emission Spectroscopy (ICP-AES)

The concentration of metal ions in solutions with hydrogels was determined by an inductively coupled plasma atomic emission spectrometer, ICP-AES (SEIKO SPS3520, Tokyo, Japan) after an appropriate dilution of samples with 0.1 M solution of HNO_3_.

### 4.3. Sample Preparation

#### 4.3.1. Preparation of CMCNF-CS and CMC-CS Hydrogels

A 3 g, 2% (*w*/*w*) dispersion of CMCNF was transferred to a petri dish 32 mm in diameter and 16 mm in height. CS powder (30 mg, 90 mg, or 120 mg) was added under stirring, and, after homogenous dispersion, GDL (70 mg) was added and stirred for ca. 30 min. The gelated mixture was allowed to stand for 24 h, and the resulting hydrogel was equilibrated in 500 mL of deionized water three times for 24 h. CMC-CS hydrogels were prepared in a similar manner. Hydrogel was placed in a round-bottom flask and dried overnight in an Eyela FDU-1200 freeze-dryer. The polymer loss was calculated as the difference between the weight of polymers used for hydrogel preparation and the dry weight of the hydrogel. The swelling ratio of a hydrogel was calculated using Equation (3):

(3) $$Q=\frac{m_{wet}-m_{dry}}{m_{dry}}\times 100\%$$

in which $m_{wet}$ and $m_{dry}$ are weights of swollen and lyophilized hydrogel, respectively.

#### 4.3.2. Adsorption of Heavy Metal Ions by CMCNF-CS and CMC-CS Hydrogels

A total of 50 mL of 10 ppm aqueous solutions of Cu^2+^, Cd^2+^, and Hg^2+^ were placed in a 50 mL tube, and the pH of each solution was adjusted to a pH of around 7. Next, about 1 g of either CMCNF-CS or CMC-CS hydrogel was added to a metal ion solution and stirred using a rotator at 20 rpm. A small volume of metal ion solution above a hydrogel was taken after different times, diluted to an appropriate concentration by a 0.1 M solution of HNO_3_, and the concentration of metal ion was measured using ICP-AES.

### 4.4. Adsorption Capacity and Kinetics Model Fitting

Adsorption capacity ($Q_{e}$) of hydrogels was calculated using Formula (4):

(4) $$Q_{e}=\frac{V\left(C_{0}-C_{t}\right)}{m}$$

in which $C_{0}$ (mmol/L) is the initial concentration of metal ion, $C_{t}$ (mmol/L) is the concentration of metal ion at time (t), V (L) is the solution volume, and m (g) is the weight of the polymers in the hydrogel adsorbent.

Equations (5) and (6) were used to fit absorption data from the pseudo-first- and pseudo-second-order absorption kinetic models, respectively.

(5) $$ln\left(Q_{e}-Q\left(t\right)\right)=lnQ_{e}-k_{1}t$$

(6) $$\frac{t}{Q(t)}=\frac{t}{Q_{e}}+\frac{1}{k_{2}Q_{e}^{2}}$$

in which Q(t) (mmol/g) is the amount of adsorbed metal ions at time t (min); $Q_{e}$ (mmol/g) is the Q(t) value at equilibrium; $k_{1}$ (min^−1^) and $k_{2}$ (g/mmol·min) are the pseudo-first- and pseudo-second-order models’ kinetic rate constants, respectively.

## Figures and Tables

**Table 1:** Comparison of swelling degrees and degrees of polymer elution of CMCNF-CS and CMC-CS hydrogels.

	CMCNF-CS Hydrogel			CMC-CS Hydrogel		
m (CS)/mg	30	90	120	30	90	120
Swelling degree in water (pH = 6.8)/%	34%	35%	43%	82%	197%	272%
Polymer elution/%	~0%	20.3%	29.3%	26.9%	56.6%	59.4%

**Table 2:** Kinetics parameters of the pseudo-first- and pseudo-second-order models for metal ion adsorption by CMC-CS and CMCNF-CS hydrogels.

Adsorbate	Pseudo-First-Order			Pseudo-Second-Order		
	k_1_	Q_e(cal)_	R^2^	k_2_	Q_e(cal)_	R^2^
	CMC-CS hydrogel					
Cd^2+^	−0.20	0.035	0.963	14.9	0.057	0.999
Cu^2+^	−0.17	0.053	0.960	2.8	0.082	0.888
Hg^2+^	−0.09	0.008	0.239	41.4	0.015	0.937
	CMCNF-CS hydrogel					
Cd^2+^	−0.11	0.030	0.823	22.3	0.050	0.999
Cu^2+^	−0.07	0.023	0.509	46.8	0.036	0.996
Hg^2+^	−0.11	0.013	0.780	19.0	0.019	0.936

## Data Availability

The data presented in this study are available on request from the corresponding author.

## Supplementary Materials

The following supporting information can be downloaded at: https://www.mdpi.com/article/10.3390/gels10090604/s1, Figure S1: FTIR spectra of CMCNF-CS90 and CMC-CS90 hydrogels; Figure S2: Results of linear fitting of Cu^2+^, Cd^2+^, and Hg^2+^ adsorption by CMCNF-CS90 and CMCNF-CS90 hydrogels using pseudo-first (A,C) and pseudo-second (B,D) kinetic models.
