# Supplementary material for: Polyion Hydrogels of Polymeric and Nanofibrous Carboxymethyl Cellulose and Chitosan: Mechanical Characteristics and Potential Use in Environmental Remediation
Source: Gels. 2024 Sep 23;10(9):604. doi: 10.3390/gels10090604 (PMC11431401; doi:10.3390/gels10090604)
Supplement: Supplementary file 1 [file gels-10-00604-s001.zip › gels-3159319-supplementary.pdf]

Supplementary Material

Polyion Hydrogels of Polymeric and Nanofibrous Carboxymethyl Cellulose and Chitosan: Mechanical Characteristics and Potential Use in Environmental Remediation

Taisei Kawate,[1] Yehao Wang,[1] Kayee Chan,[1] Nobuyuki Shibata,[2] Yuya Doi,[3,4] Yuichi Masubuchi,[3] Anatoly Zinchenko[1] *

[1] Graduate School of Environmental Studies, Nagoya University, Nagoya, 464-8601 Japan; zinchenko@urban.env.nagoya-u.ac.jp
[2] Nagoya Municipal Industrial Research Institute, Nagoya, 456-0058, Japan; shibata.nobuyuki@nmiri.city.nagoya.jp
[3] Graduate School of Engineering, Nagoya University, Nagoya, 464-8601 Japan; masubuchi@nagoya-u.jp
[4] Graduate School of Organic Materials Science, Yamagata University, Yonezawa, 992-8510 Japan; ydoi@yz.yamagata-u.ac.jp

1. FTIR spectra of CMCNF-CS90 and CMC-CS90 hydrogels.

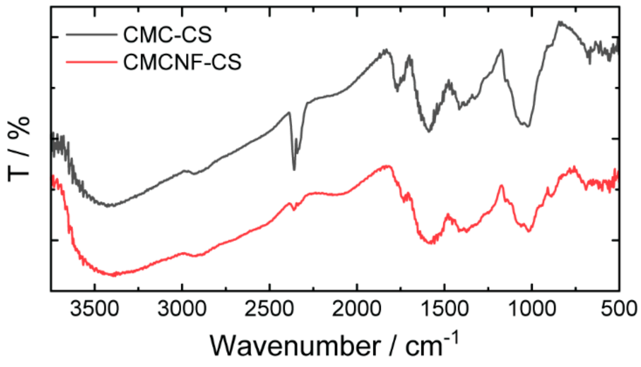

Figure S1. FTIR spectra of freeze-dried CMCNF-CS90 and CMC-CS90 hydrogels.

2. Adsorption kinetics fitting

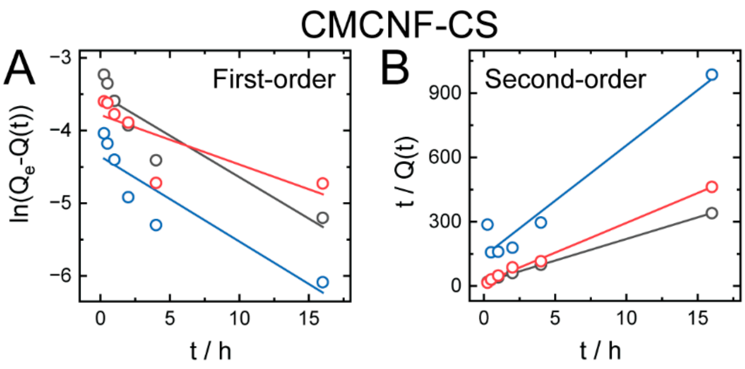

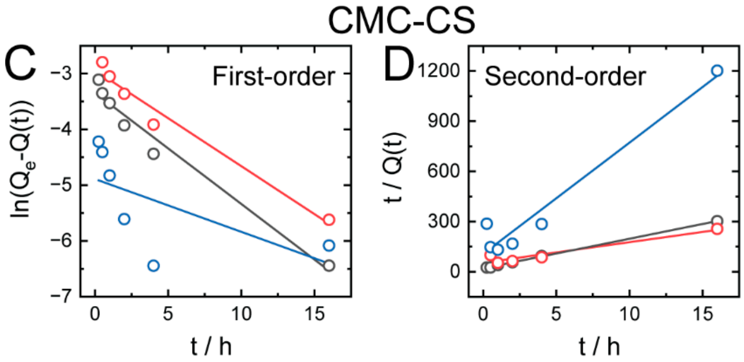

Figure S2. Results of linear fitting of Cu^{2+}, Cd^{2+}, and Hg^{2+} adsorption by CMCNF-CS90 and CMCNF-CS90 hydrogels using pseudo-first (**A, C**) and pseudo-second (**B, D**) kinetic models.
